# Effects of dietary zinc on the gut microbiome and resistome of the gestating cow and neonatal calf

**DOI:** 10.1186/s42523-024-00326-3

**Published:** 2024-07-19

**Authors:** Mary Jane Drake, Scott G. Daniel, Linda D. Baker, Nagaraju Indugu, Kyle Bittinger, Charlene Dickens, Joseph P. Zackular, Dipti Pitta, Laurel E. Redding

**Affiliations:** 1 Clinical Studies – New Bolton Center, School of Veterinary Medicine, University of Pennsylvania, Kennett Square, PA USA; 2 Division of Gastroenterology, Hepatology and Nutrition, Children’s Hospital of Philadelphia, Philadelphia, PA 19104 USA; 3 Division of Protective Immunity, Children’s Hospital of Philadelphia, Philadelphia, PA 19104 USA; 4 Department of Pathology and Laboratory Medicine, Perelman School of Medicine, University of Pennsylvania, Philadelphia, PA 19104 USA; 5 Institute for Immunology, Perelman School of Medicine, University of Pennsylvania, Philadelphia, PA 19104 USA

**Keywords:** Zinc, Dairy cattle, Calves, Microbiome, Resistome, Gestation

## Abstract

Zinc is an essential trace element required in the diet of all species. While the effects of zinc have been studied in growing calves, little is known about the effect of zinc on the microbiota of the gestating cow or her neonatal calf. Understanding factors that shape the gut health of neonatal animals and evaluating the effect of dietary supplements in adult gestating animals is important in promoting animal health and informing feeding practices. The aims of this study were to determine the effect of dietary zinc on the microbiota and resistome of the gestating cow and calf. Gestating cows received standard (40 ppm) or high (205 ppm) dietary zinc levels from dry off to calving. Fecal samples were collected from cows upon enrollment and at calving and from neonatal calves. Fecal samples underwent 16S rRNA sequencing and a subset also underwent shotgun metagenomic sequencing. The effect of zinc supplementation on the diversity and composition of the cow and calf microbiome and resistome was assessed. Alpha and beta diversity and composition of the microbiota were significantly altered over time but not by treatment in the cows, with alpha diversity decreasing and 14 genera found at significantly higher relative abundances at calving compared to enrollment. Levels of 27 antimicrobial resistance genes significantly increased over time. Only a small number of taxa were differentially expressed at calving in treatment and control groups, including *Faecalibacterium*, *Bacteroides*, *Turicibacter*, and *Bifidobacterium pseudolongum*. No effect of the dam’s treatment group was observed on the diversity or composition of the neonatal calf microbiota. The calf resistome, which was relatively rich and diverse compared to the cow, was also unaffected by the dam’s treatment group. The impact of high levels of dietary zinc thus appeared to be minimal, with no observed changes in alpha or beta diversity, and few changes in the relative abundance of a small number of taxa and antimicrobial resistance genes.

**Supplementary Information:**

The online version contains supplementary material available at 10.1186/s42523-024-00326-3.

## Introduction

The gut microbiota plays a crucial role in maintaining the health and physiological functions of animals, impacting various aspects such as nutrient metabolism, immune response, and overall well-being. In recent years, there has been increasing interest in understanding the intricate relationship between dietary factors and the composition and function of the gut microbiota in livestock species, especially dairy cows.

Metals are essential micronutrients for all of life, playing a central role in cellular functions for both prokaryotic and eukaryotic cells. The heavy metal zinc acts as a cofactor for numerous enzymes involved in cellular processes, immune function, and oxidative stress regulation. Extensive research has demonstrated that zinc supplementation in livestock diets can enhance growth, reproductive performance, and overall health [1–6]. Despite the importance of metals for survival, excess metals are toxic to cells, suggesting that high levels of dietary metals may impact the delicate ecology in the gut. Emerging evidence suggests that dietary zinc may modulate the composition of the gut microbiota in various animal species [7, 8]. Heavy metals such as zinc can also select for antimicrobial resistance, often through co-localization of metal resistance genes and antimicrobial resistance genes [9–11].

During gestation, dairy cows undergo significant physiological changes to support fetal development and prepare for lactation. These alterations can influence the cow’s metabolism, nutrient requirements, and overall gut health. Therefore, understanding the potential influence of dietary zinc on the gut microbiota and resistome of gestating dairy cows and their calves and the resultant effect on their susceptibility to opportunistic gastrointestinal infections is critical. While the effect of dietary zinc has been extensively investigated in growing calves and adult beef cattle [12–17], little is known about its impact in gestating dairy cows and their calves. The aim of this study was to assess the impact of high levels of dietary zinc supplementation on the health and composition of the gut microbiota and resistome in gestating dairy cows and their calves. We hypothesized that high levels of dietary zinc would induce notable changes in the gut microbial community composition, potentially affecting the overall health status of the cows.

## Methods

### Feeding trial

In total, sixty-four Holstein cows (approximate weight 550–700 kg) from the University of Pennsylvania’s Marshak Dairy were enrolled in the feeding trial at dry-off (approximately 6 weeks prior to calving) and randomized to receive a control diet with standard zinc levels (40 ppm) or a high zinc (205 ppm) treatment diet until calving. The feeding trial was performed between February and June 2022. Primiparous and multiparous cows (parity 2–5) were eligible, with the average age and parity being 3.1 ± 1.3 years and 2.1 ± 1.2 lactations, respectively (Table 1). Randomization yielded similar numbers of primiparous (control *n* = 12, zinc *n* = 13) and multiparous (control *n* = 21, zinc *n* = 18) animals between the two treatment groups. In addition to standard dry cow rations (described in Supplementary Tables 1 and 2), cows in both groups were fed a daily top-dressing of 0.113 kg of corn distiller’s grain. No supplemental zinc was added to the diet of the cows in the control group (*N* = 33), which were fed the standard dietary amount recommended by the National Research Council (40 ppm) [18]. For the treatment group (*N* = 31), zinc hydroxychloride was added to the top dress at a level of 205 ppm, or five times the level fed to the control cows, which is well below the maximum tolerable amount (500 ppm). Multiparous cows received intramammary penicillin and dihydrostreptomycin (Quartermaster, US Vet Inc., Paramus, NJ) as part of a standard dry off protocol but did not consume or receive injectable antibiotics during the feeding trial. Fecal samples were collected per rectum of cows at enrollment (*N* = 64) and at calving (*N* = 64), and from their calves (*N* = 60 total) no later than 3 days after calving (Table 1). Samples from four of the dam’s calves were not collected or lost in transit. Calves received 4 quarts of colostrum within 4 h of birth, followed by 2 quarts of pasteurized waste milk twice daily for subsequent feedings. Samples were stored at -80 °C until further processing. The study protocol was approved by the University of Pennsylvania Institutional Animal Care and Use Committee (Protocol #: 807178).

**Table 1:** Study subject parity, age, duration in feeding trial, and timing of calf’s sample collection

	**Control**	**Zinc Supp**	**p-value**
**Lactation (parity)**			
1	12	13	
2	10	8	
3	6	6	
4	3	3	
5	2	1	
Total	33	31	0.36^a^
Mean	2.2	2.1	
SD	1.2	1.2	
**Age (years)**			
Mean	3.2	3.1	0.69^b^
SD	1.4	1.3	
**Duration in Feeding Trial (days)**			
Mean	42.1	42.9	0.66^b^
SD	10.0	10.5	
Range	16-60	18-66	
**Age of Calf Sample Collection (days)**			
0	12	13	
1	12	7	
2	6	6	
3	1	3	
Total	31	29	0.49^a^
Mean	0.9	1.0	
SD	0.8	1.1	

^a^Chi-square test, ^b^Two-tailed t-test

### 16s rRNA gene sequencing

DNA was extracted from approximately 200 mg of stool using the Qiagen DNeasy PowerSoil Pro kit. Extracted DNA was quantified with the Quant-iT PicoGreen Assay Kit. Barcoded PCR primers annealing to the V1-V2 region of the 16S rRNA gene were used for library generation (forward primer: 5’-AGAGTTTGATCCTGGCTCAG-3’; reverse primer: 5’-TGCTGCCTCCCGTAGGAGT-3’). PCR reactions were carried out in duplicate using Q5 High-Fidelity DNA Polymerase (NEB, Ipswich, MA). Each PCR reaction contained 0.5 μm of each primer, 0.34 U Q5 Pol, 1X Buffer, 0.2 mM dNTPs, and or 5.0 μl DNA in a total volume of 50 μl. Cycling conditions were as follows: 1 cycle of 98 °C for 1 m; 20 cycles of 98 °C for 10 s, 56 °C for 20 s, and 72 °C for 20 s; 1 cycle of 72 °C for 8 m. After amplification, duplicate PCR reactions were pooled and then purified using a 1:1 volume of SPRI beads. DNA in each sample was then quantified using PicoGreen and pooled in equal molar amounts. The resulting library was sequenced on the Illumina MiSeq using 2 × 250 bp chemistry. Extraction blanks and DNA free water were subjected to the same amplification and purification procedure to allow for empirical assessment of environmental and reagent contamination. Positive controls, consisting of eight artificial 16S gene fragments synthesized in gene blocks and combined in known abundances, were also included.

### Shotgun metagenomic sequencing

A subset of the samples was subject to shotgun metagenomic sequencing to further characterize taxonomy and antimicrobial resistance within the study populations. Financial limitations prohibited sequencing of the entire sample set. Twenty cows each from the control and treatment groups were included as well as 32 of their calves (16 per study group). Selected cows had a dry period and enrollment in the trial of at least 35 days, had a matching calf sample, and were balanced for parity (8 primiparous and 12 multiparous per treatment). Calf samples were balanced for the day of sample collection between dam’s treatment groups.

DNA extracted from 200 mg of stool were quantified using the Quant-iTTM PicoGreen dsDNA assay kit (Thermo Fisher Scientific) before library generation. Shotgun libraries were generated from 7.5 ng DNA using Illumina DNA Prep Library Prep kit and IDT for Illumina unique dual indexes at 1:4 scale reaction volume. Library success was assessed by Quant-iT PicoGreen dsDNA assay and samples with library yields < 1 ng/μl were re-prepped as needed. After all samples for a given pool are prepped, an equal volume of library was pooled from every sample and then the pool was sequenced using a 300 cycle Nano kit on the Illumina MiSeq. Libraries will then be repooled based on the demultiplexing statistics of the MiSeq Nano run. Final libraries were QCed on the Agilent BioAnalyzer to check the size distribution and absence of additional adaptor fragments. Libraries were sequenced on an Illumina Novaseq 6000 v1.5 flow cell, producing 2 × 150 bp paired-end reads. Extraction blanks and nucleic acid-free water were processed along with experimental samples to empirically assess environmental and reagent contamination. A laboratory-generated mock community consisting of DNA from *Vibrio campbellii* and Lambda phage was included as a positive sequencing control.

### Bioinformatics processing

The QIIME2 pipeline was used to process and analyze 16S rRNA sequencing data. Samples were demultiplexed using q2-demux and denoised using Dada2. Sequences were aligned using maaft and phylogenetic trees were reconstructed using fasttree. Shannon alpha diversity, weighted UniFrac and Bray-Curtis beta diversity metrics were estimated using q2-coremetrics-diversity after samples were rarefied, and p-values were adjusted for multiple hypothesis testing using Benjamini-Hochberg (B-H) false discovery rate (FDR) corrections. Taxonomy was assigned to sequences using q2-feature-classifier classify-sklearn against the Silva reference database. ASVs with less than 1% average relative abundance across all samples were removed.

Shotgun metagenomic data were analyzed using Sunbeam, a user-extendable bioinformatics pipeline that we developed for this purpose [PMID 30902113]. Quality control steps were performed by the default workflows in Sunbeam, which are optimized to remove host-derived sequences and reads of low sequence complexity. The abundance of bacteria was estimated using Kraken [PMID 24580807]. Reads were mapped to the CARD database [PMID 36263822] using Diamond [PMID 25402007] to estimate the abundance of anti-bacterial resistance genes (ARGs). To account for different gene lengths, ARGs were normalized to reads per kilobase gene length per million mapped reads (RPKM).

### Statistical analysis

The Wilcoxon rank sum test or Kruskal-Wallis test, as appropriate, was used to compare measures of alpha diversity over time and between treatment groups. A nonparametric permutational multivariate ANOVA (PERMANOVA) was implemented to evaluate the effects of collection time, treatment group, time in trial, and parity on the overall microbiota community composition as measured by the weighted pairwise UniFrac distance. Generalized linear models were used to assess the effect of time and treatment group on relative abundances of different bacterial taxa. Analysis of the composition of the microbiome (ANCOM) was performed to determine whether certain taxa were differentially expressed over time and across groups.

For shotgun data, the abundance of taxa was analyzed at a community level using pairwise distance between samples (Bray-Curtis and Jaccard) and visualized with Principal Coordinates Analysis. Community-level differences between sample groups were assessed using the PERMANOVA test. Linear models were used to detect differences in log_2_-transformed ARG and taxon abundance between sample groups (i.e. zinc supplementation and sampling time). P-values from multiple testing procedures were corrected to control for a specified false discovery rate (0.05). Correlation analysis between bacterial taxa (relative abundance) and ARG abundance (RPKM) was performed using Pearson’s product moment correlation coefficient and the cor.test function in R used to determine significance after false discovery rate correction using the Benjamini-Hochberg method. To account for large differences in taxa abundance between cows and calves, correlation analysis of combined cow and calf samples was modified and performed on the residuals from linear models of taxa and ARG abundance.

## Results

### Dietary zinc feeding trial and the gestating cow gut microbiome

Dietary supplementation is a frequently used strategy to improve growth and the health of growing calves and adult cattle. However, little is known about the impact of high levels of dietary zinc on the gut microbiota. To systematically determine this, a total of 64 cows were enrolled in this study, including 33 in the control group and 31 in the treatment group. Twenty-five, 18, 12, 6 and 3 cows were in their first, second, third, fourth, and fifth lactation, respectively. The overall distribution of parity within the trial is reflective of the study herd demographics, and cows were randomized to treatment group at enrollment. There was no difference in distribution of parity (Chi-square test, *p* = 0.355) or days in the trial (t-test, *p* = 0.655) between treatment groups (Table 1). The mean (SD) time spent in the feeding trial was 42.9 (10.5) days for the treatment diet and 42.1 (10.0) days for the control diet.

To define the impact of dietary zinc supplementation on the succession and development of the gut microbiota in the gestating cow, we performed amplicon-based sequencing of the 16S rRNA gene from longitudinally collected fecal samples. A total of 9,491,209 raw reads were generated from a total of 128 samples, with an average of 75,327 ± 13,032 (mean ± SD) reads per sample. Six experimental samples had less than 100 reads per sample, and they were dropped from the analysis. This produced a total of 4,039,443 amplicon sequence variants (ASVs). Representative sequences from the ASV were assigned to 18 distinct bacterial phyla, 3 of which could not be classified. A total of 247 genera categories were observed in this study, 32 (12.9%) of which were unclassified to either phylum, class, order, or family, and 71 (28.7%) of which could not be identified to the genus level.

To complement the results obtained with 16S rRNA gene sequencing at a more granular level and to characterize the effect of zinc supplementation on antimicrobial resistance gene (ARG) abundance and diversity, a subset of samples was subject to shotgun metagenomic sequencing. In total, samples from 40 cows (*N* = 20 per treatment group at enrollment and calving) and 32 calves (*N* = 16 per dam’s treatment group) were analyzed. Sequencing generated 690,439,341 total reads from 110 samples with an average of 6,276,721 ± 2,660,931 (mean ± SD) reads per sample. Two calf samples with < 15,000 reads were excluded from analysis. Despite the robust metagenomic sequencing coverage, fewer relative taxonomic assignments were made using Kraken (11.2% of cow reads, 84.4% of calf reads) relative to 16S rRNA sequencing (90.1% of reads) due to limited rumen-associated annotated taxa.

**Fig. 1:**
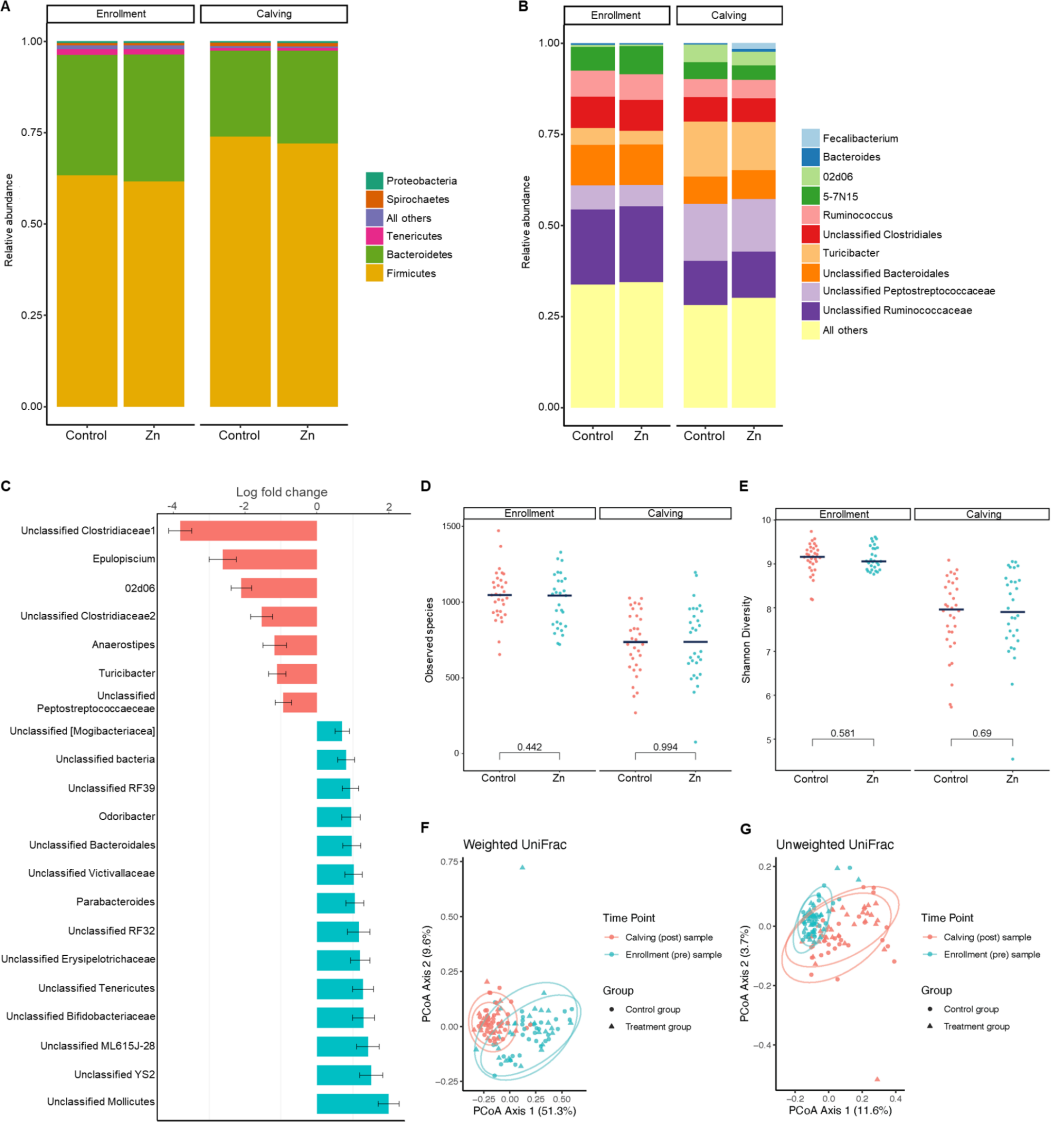
Taxonomic and bacterial diversity analysis of gestating cows enrolled in a zinc dietary trial. (**A**,**B**) Mean relative abundances of the predominant bacterial phyla (**A**) and genera (**B**) in the fecal samples of cows on a control diet (Control) or diet supplemented with zinc (Zn) during the dry period. Samples were collected at trial enrollment and approximately 6 weeks later at calving and subject to 16S rRNA gene sequencing. (**C**) Log-fold changes in bacterial taxa at calving relative to enrollment. Red bars indicate taxa that were seen at higher levels at calving. Blue bars indicate taxa that were seen at higher levels at enrollment. (**D**,**E**) Alpha diversity was calculated using bacterial species richness (**D**) and Shannon diversity (**E**). Black horizontal line indicates median values. The number above bracket indicates p-value (Wilcoxon rank sum test). (**F**,**G**) Beta diversity measurements are illustrated using principal coordinate plots of weighted (**F**) and unweighted (**G**) paired UniFrac distances

The most commonly observed phyla in the cow fecal samples were Firmicutes and Bacteroidetes (Fig. 1A). Significant differences in the proportion of all phyla were observed between enrollment and calving (*p* < 0.001) but not between treatment groups. A similar trend was observed for genera (Fig. 1B), with significant changes seen for most genera between enrollment and calving but not between treatment groups. Metagenomic sequencing of the cow samples aligned with results obtained with 16S rRNA gene sequencing, with dominant phyla being Firmicutes (41%), Proteobacteria (17%), Bacteroidetes (15%), and Actinobacteria (12%) (Supplemental Fig. 1). ANCOM results showed that 7 genera were significantly more abundant at calving related to enrollment, whereas 14 were found at significantly higher relative abundance at enrollment related to calving (Fig. 1C). These abundance differences occurred consistently in both the treatment and control groups. However, there were two genera for which changes in relative abundance occurred differentially for treatment and control groups. *Faecalibacterium* was not detected at either enrollment or calving in the control group (no change), whereas a 46-fold increase in abundance was seen between enrollment and calving for the treatment group (light blue bar, Fig. 1B). Similarly, *Bacteroides* levels increased at calving in the treatment group (2.16-fold change in abundance) and decreased in the control group (0.75-fold change) (dark blue bars, Fig. 1B). Shotgun metagenomic sequencing also documented an increase in the Firmicute *Turicibacter sp*. H121 over time (control *p* < 0.001, treatment *p* = 0.036) but no significant change across treatment groups (Supplemental Fig. 2). This was also seen at the genus level in our 16S rRNA gene sequencing (Fig. 1B, C). Levels of the Actinobacteria *Bifidobacterium pseudolongum* significantly increased in the zinc supplemented group (*p* = 0.026) over time, and while not significant, trended similarly in the control group over time (*p* = 0.19) (Supplemental Fig. 2).

There was a statistically significant difference in the number of observed ASVs (i.e., species richness) between enrollment and calving (*p* < 0.001) but not between the zinc treatment groups (*p* = 0.994) (Fig. 1D). A similar trend was observed for the Shannon diversity index (enrollment vs. calving: *p* < 0.001, control vs. zinc supplemented group: *p* = 0.69) (Fig. 1E). Neither parity nor time in trial affected either the species richness (*p* = 0.267 and *p* = 0.477, respectively) nor Shannon diversity (*p* = 0.694 and *p* = 0.609, respectively).

As was seen for alpha diversity, a significant difference in beta diversity was observed over time for both weighted (*p* < 0.001) (Fig. 1F) and unweighted Unifrac (*p* < 0.001)(Fig. 1G) metrics but not for treatment group (*p* = 0.626 and *p* = 0.524, respectively), parity (*p* = 0.447 and *p* = 0.105, respectively), or number of days in the trial (*p* = 0.355 and *p* = 0.160, respectively).

### Gestating cow resistome

**Fig. 2:**
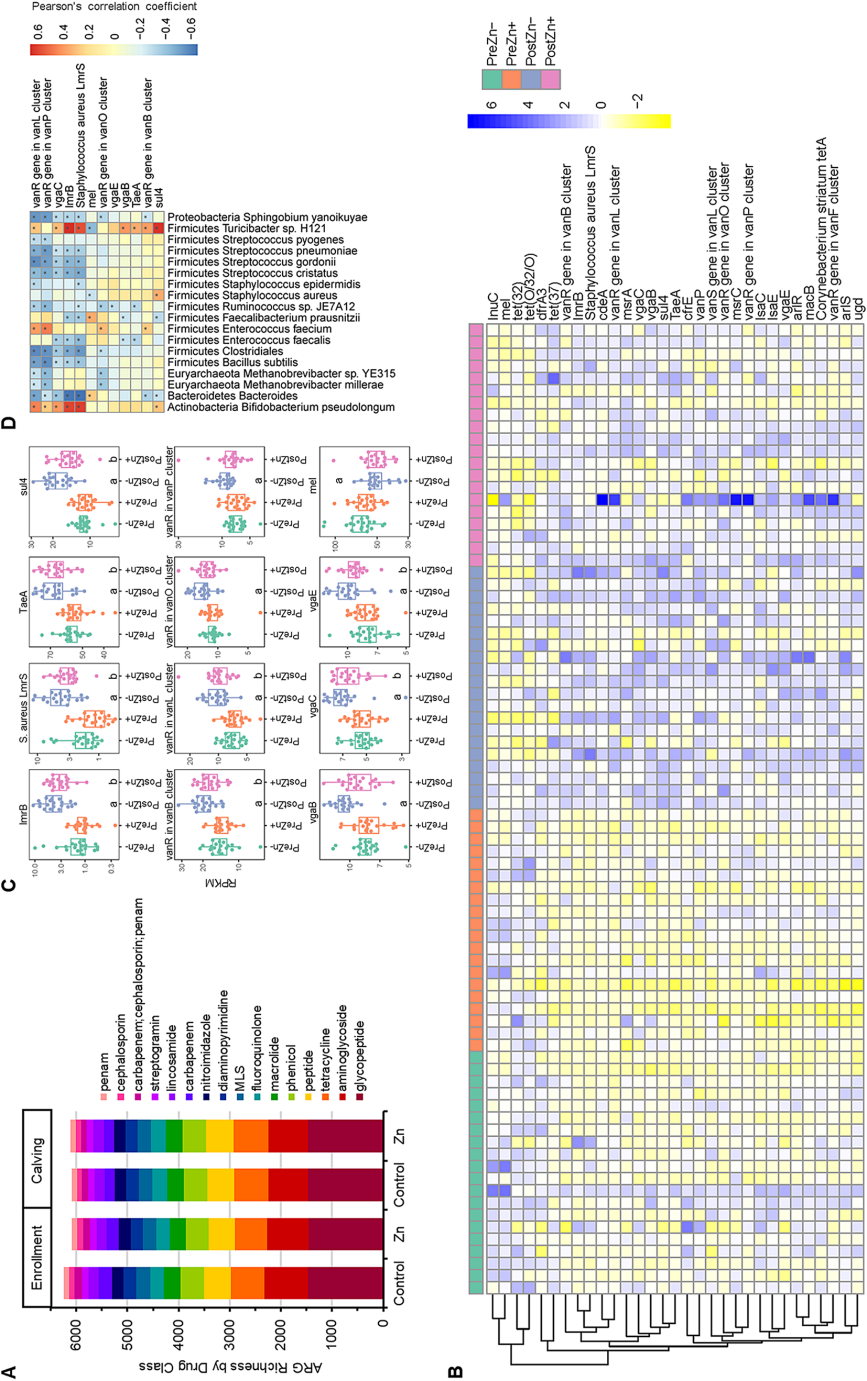
Antibacterial resistome in gestating cows enrolled in a zinc dietary trial. (**A**) Metagenomic sequencing was used to characterize the fecal resistome in gestating cows supplemented with zinc (Zn) or fed a control diet (Control) during the dry period (Enrollment, Calving). The number of unique antimicrobial resistance genes (ARG) per drug class per study group is depicted. Drug classes with richness greater than 100 for an individual study group were included. (**B**) Relative abundance heatmap of ARGs that significantly differed between enrollment and calving in gestating cows in at least one study population. Reads per Kilobase per Million mapped reads (RPKM) were scaled for each ARG to generate the depicted z-score. Blue indicates higher abundances while yellow indicates lower relative abundance. Each column represents individual cow sample either at enrollment (Pre) or calving (Post) for control (Zn-) and zinc supplemented (Zn+) treatment groups. (**C**) Relative abundance of ARGs that were significantly different (adjusted p-value < 0.05) over time for control (**a**) and zinc supplemented (**b**) groups are depicted. Box plots illustrate RPKM values by study group (Zn- or Zn+) at enrollment (Pre) and calving (Post). (**D**) Heatmap depicts correlation analysis using Pearson’s product moment correlation coefficient between relative abundance of bacterial taxa and ARG abundance (RPKM) for ARGs that significantly changed over time in one or both cow treatment groups (Zn, control) and were highlighted in (**D**). Boxes with asterisk indicate significant correlations after false discovery rate correction (*p* < 0.05)

Heavy metals such as zinc can select for antimicrobial resistance in livestock species through co-selection with metal resistance genes on plasmids and mobile genetic elements [9–11]. Shotgun metagenomic sequencing was used to investigate the effect of zinc on antimicrobial resistance gene abundance and diversity. Using the Comprehensive Antibiotic Resistance Database (CARD) as a reference [19], 195 ARGs were found to be present in 99% of cow samples. Genes conferring resistance to glycopeptides, aminoglycosides, tetracyclines, peptides, and phenicol antibiotics were most commonly observed (Fig. 2A). As expected, ARG abundances were similar between treatment and control groups at enrollment. Between enrollment and calving, levels of 27 ARGs significantly increased, including 13 in the zinc supplemented group, five in the control group, and nine shared by both treatment groups (Fig. 2B; Table 2). Nine of the 27 ARGs that significantly increased over time conferred resistance to multiple antibiotic drug classes. Additionally, seven of ARGs conferred resistance to glycopeptide antibiotics (*vanP*, *vanRB*, *vanRF*, *vanRL*, *vanRO*, *vanRP*, *vanSL*) by altering cell wall peptidoglycan precursors, thus preventing glycopeptide binding. Two ARGs significantly decreased in the control group over time (*linC*, *meI*), while two ARGs conferring tetracycline resistance [*tet(32)*, *tet(O/32/O)*] decreased in the high-zinc group over time. For all of the ARGs where there was a significant change in abundance over time, a non-statistically significant trend appeared: relative abundance levels were lower in the treatment groups than in the control groups (Fig. 2C).

**Table 2:** Antimicrobial resistance genes (ARGs) that significantly changed over time by treatment group

	Zinc Supplemented			Control	
ARG	Estimate^1^	Adjusted *p*-value^2^		Estimate	Adjusted *p*-value
arlR	7.1509	0.2255		10.9530	0.0214
arlS	2.9502	0.0355		2.6930	0.0646
cdeA	3.5636	0.0304		2.4459	0.2217
cfrE	0.8622	0.0308		0.2034	0.9101
dfrA3	2.1163	0.0444		0.6668	0.8339
lmrB	2.3599	0.0010		2.7245	0.0001
lmrS	2.1961	0.0039		2.4724	0.0009
lnuC	-18.7741	0.0601		-23.1099	0.0126
lsaC	1.4686	0.0126		0.7231	0.4155
lsaE	2.7616	0.0458		0.8865	0.8282
macB	8.5596	0.0103		4.8985	0.2642
mel	-9.4694	0.2096		-18.2777	0.0016
msrA	0.6374	0.0041		0.3842	0.1743
msrC	1.1886	0.0227		-0.0343	0.9997
pmrE	13.3181	0.0069		8.6881	0.1380
sul4	4.4135	0.0003		7.4272	1.93E-09
TaeA	12.0411	0.0002		10.0349	0.0022
tet(32)	-2.3022	0.0339		-2.0473	0.0726
tet(37)	4.2348	0.0320		0.6573	0.9723
tet(O/32/O)	-1.0878	0.0209		-0.8546	0.1012
tetA	3.4244	0.0098		1.1255	0.7141
vanP	0.8327	0.1211		1.0066	0.0405
vanRB	3.8056	0.0063		5.2552	0.0001
vanRF	7.8844	0.0380		5.3077	0.2623
vanRL	3.5750	0.0008		3.2982	0.0022
vanRO	2.0287	0.1139		3.1476	0.0040
vanRP	1.7231	0.3115		2.6523	0.0443
vanSL	0.6593	0.0278		0.7794	0.0063
vgaB	1.1528	0.1145		2.1650	0.0003
vgaC	1.0612	0.0389		1.6303	0.0004
vgaE	1.3739	0.0306		2.1188	0.0002

^1^Linear mixed effect models estimated change of log_2_ transformed RPKM values between study groups

^2^P-value adjusted for post-hoc multiple comparisons with Tukey’s method

Correlation analysis was performed using Pearson’s correlation coefficient to investigate the putative taxonomic origins of the ARGs that significantly changed over time (Fig. 2D). *Bifidobacterium pseudolongum* and *Turicibacter* sp. H121 had significant positive correlations with several shared ARGs including *vanRL*, *vgaC*, *lmrB*, *lmrS*, and *sul4*. These bacterial species had an approximately 4 to 10-fold increase in relative abundance between enrollment and calving in both control and zinc supplemented groups (Supplemental Fig. 2), and their relative increases may explain the increased abundance of these specific ARGs. Additional positive correlations were also found with *Enterococcus faecium, Fecalibacterium prausnitzii*, and *Staphylococcus aureus.* Many significant negative correlations were also found, indicating bacterial species that were less likely to harbor those ARGs. Taken together, these results suggest that several diverse species of bacterial taxa contribute to the changes observed in the resistome over time.

### Neonatal calf gut microbiome

Because the gut microbiota of the neonatal calf is shaped in part by the environment into which it is born and by the exposures of its dam [20, 21], we investigated the gut microbiota of 60 calves born to the cows enrolled in the feeding trial using 16S rRNA and shotgun metagenomic sequencing. Fecal samples collected on the day of birth, and at 1, 2 and 3 days of age accounted for 42%, 32%, 20% and 6.7% of samples, respectively. There was no difference in when the samples were collected based on the dam’s treatment group (Chi-square test, *p* = 0.492, Table 1). For 16S rRNA sequencing, a total of 1,828,327 raw reads were generated from a total of 41 calf samples, with an average of 44,593 ± 15,508 (mean ± SD) reads per sample. Fifteen calves had less than 1000 (ranged between 0 and 675) reads per sample, and the sequencing depth for 4 other experimental samples had ranged between 1014 and 3588, and they were dropped from the analysis. All excluded samples were collected within the first 24 h of life. Colonization and development of the gut microbiome occurs during and shortly after parturition. However, meconium generally does not contain much bacterial DNA [22] and rather contains host-derived waste products accumulated within the gastrointestinal tract *in utero*. This can explain why so many early calf samplings had low DNA yields and insufficient sequence coverage. The 41 samples passing quality control produced a total of 1,151,032 amplicon sequence variants (ASVs). Representative sequences from the ASVs were assigned to 11 bacterial phyla. A total of 172 genera were observed in this study. A subset of calf samples (*n* = 32) also underwent shotgun metagenomic sequencing to characterize the calf resistome. From the sequencing, a total of 728 million raw reads were generated with an average sequencing quality score of 36 (Phred33). After quality control and removal of host reads, 637 million reads were left for analysis.

**Fig. 3:**
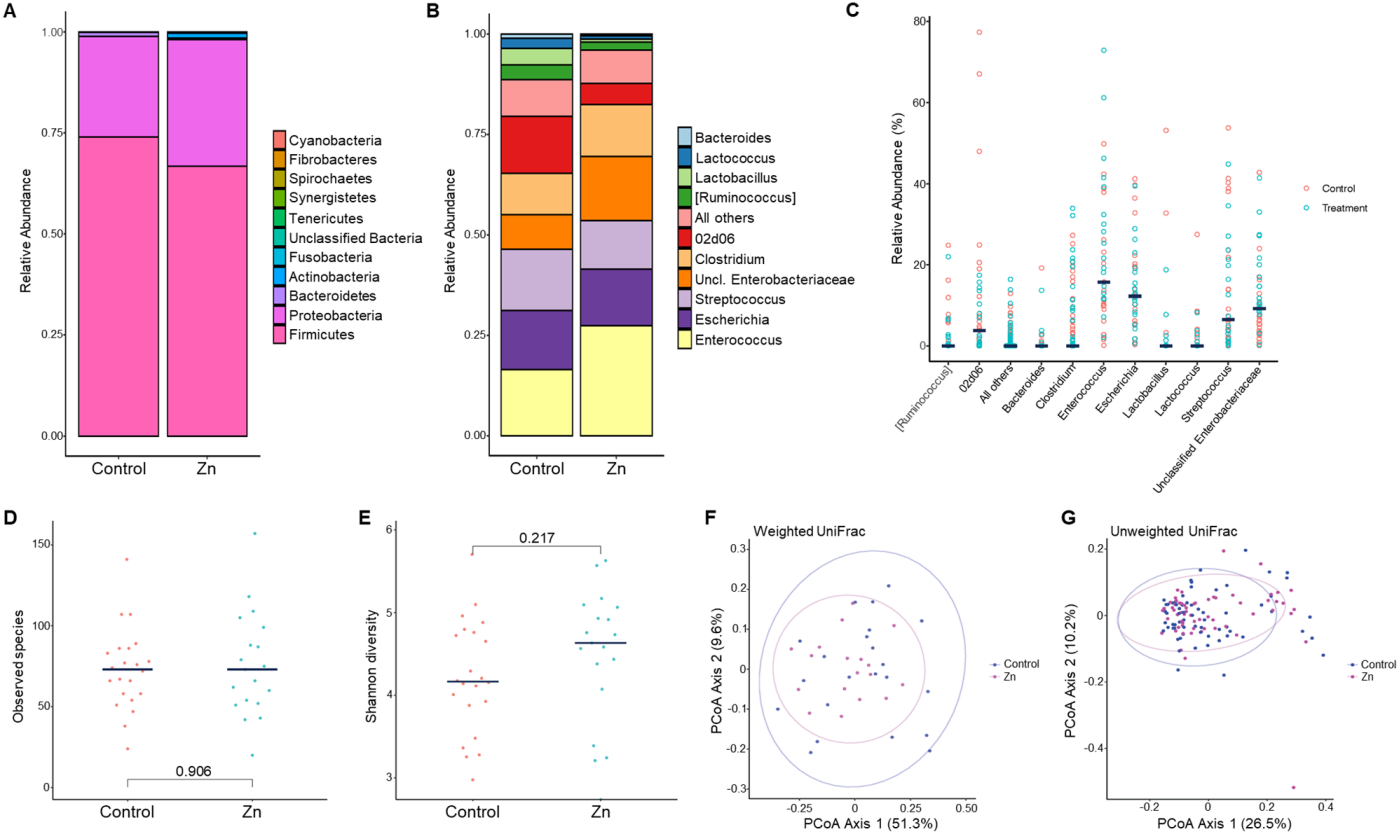
Taxonomic and bacterial diversity analysis of calves born to dams in a dietary zinc trial. (**A**, **B**) Mean relative abundances of predominant bacterial phyla (**A**) and genera (**B**) in fecal samples of neonatal calves. Dams were supplemented with zinc (Zn) or received a control diet (Control) during the last 6 weeks of gestation. Calf samples were collected within the first three days of birth. (**C**) Comparison of most abundant genera in calves by treatment group. Relative abundances of the ten most abundant genera in calves of cows enrolled in the controlled feeding trial. Horizontal black bars represent median levels. Coloring represents the treatment group of the calf’s dam. (**D**, **E**). Alpha diversity measurements of bacterial species richness (**D**) and Shannon diversity (**E**) from calves. Black horizontal lines indicate median values. Number above bracket indicates p-value (Wilcoxon rank sum test). (**F**, **G**) Beta diversity measurements were used to compare bacterial communities across treatment groups and illustrated using principal coordinate plots of weighted (**F**) and unweighted (**G**) UniFrac distances

The most common phyla were Proteobacteria and Firmicutes (Fig. 3A). No significant difference in the proportions of any phyla were observed across treatment groups. The most commonly observed genera were *Enterococcus, Escherichia* and *Streptococcus* (Fig. 3B). ANCOM revealed no significant difference in genera abundance by treatment group, though calves born to dams in the treatment group had non-statistically significantly increased levels of *Enterococcus* and *Enterobacteriaceae* (yellow and dark orange bars, respectively, Fig. 3B), and decreased levels of *Lactobacillus*, *Lactococcus*, and *Bacteroides* (light green, dark blue, and light blue bars, respectively, Fig. 3B).

In general, there was moderate variability in the relative abundances of each genera across all calves (Fig. 3C). Shotgun metagenomic sequencing of fecal samples from neonatal calves revealed similar findings to 16S rRNA sequencing. The microbiome was dominated by Proteobacteria and Firmicutes, with Proteobacteria tending to be higher (*p* = 0.064) and Firmicutes lower (*p* = 0.064) in calves from zinc-supplemented dams compared to controls (Supplemental Fig. 1). *Escherichia coli* was found at a high relative abundance in calf samples (0.5–29% per sample), which is consistent with its typical early colonization of the gastrointestinal tract [23]. Other earlier gut colonizers *Enterococcus faecalis*, *Enterococcus faecium*, and *Clostridium perfringens* also had variable relative abundance ranging from 0.02 to 65%, 0.03–8.7%, and 0.03–10.8%, respectively, which was similar to results found at the genus level with 16S rRNA analysis. There were no significant differences between treatment groups at the species level.

No significant differences in species richness (*p* = 0.906) (Fig. 3D), Shannon diversity (*p* = 0.217) (Fig. 3E) or in beta diversity as measured by weighted (Fig. 3F) and unweighted (Fig. 3G) Unifrac (*p* = 0.224 and *p* = 0.240, respectively) were observed between calves from different treatment groups.

### Calf gut resistome

**Fig. 4:**
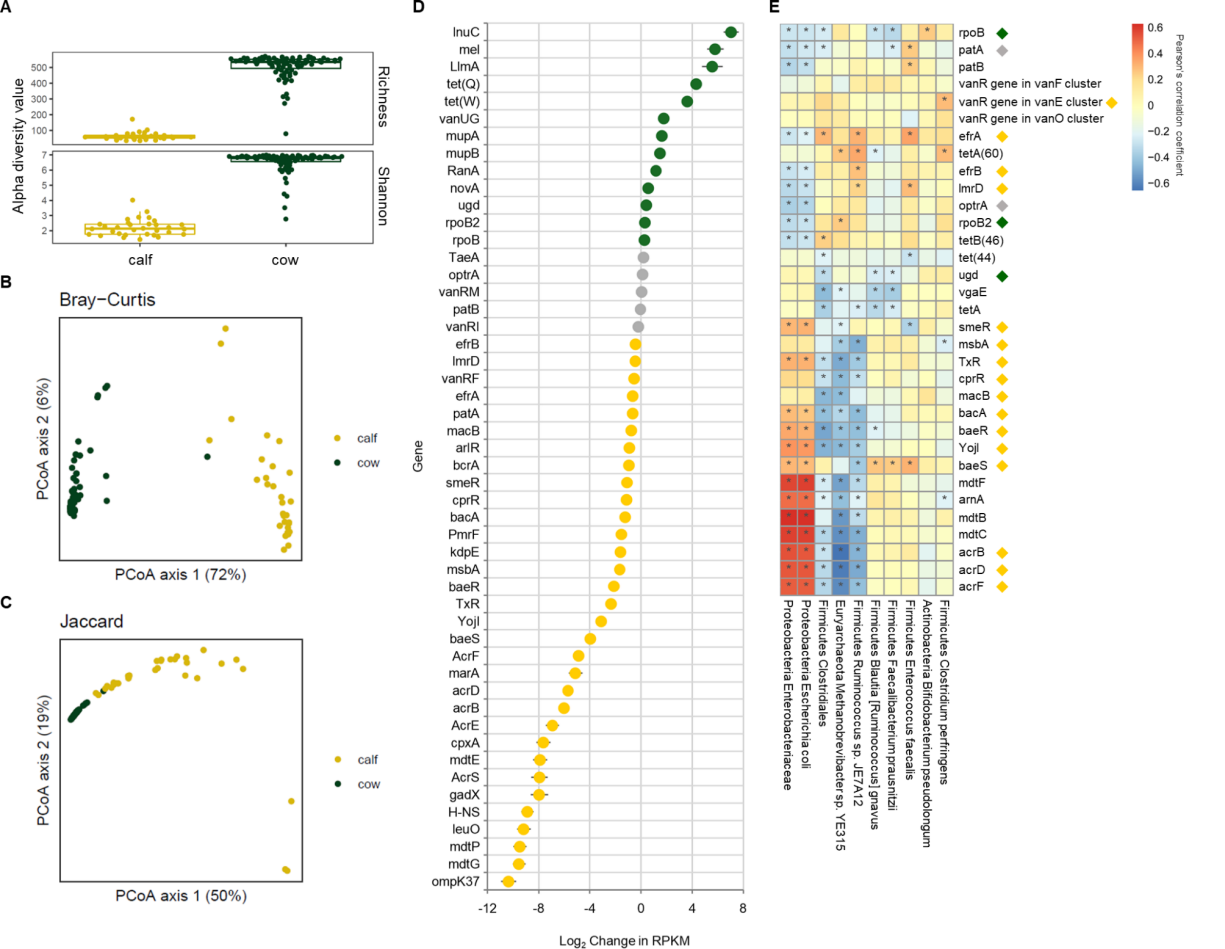
Comparison of fecal bacterial diversity and resistome in gestating cows enrolled in a dietary zinc trial and their calves. (**A**) Alpha diversity of fecal samples (richness and Shannon diversity) from cows and their calves. (**B**, **C**) Species composition from fecal samples of cows and calves collected during the dietary zinc feeding trial. Bray-Curtis (**B**) and Jaccard (**C**) distances are represented using 2D Principal Coordinate Analysis. Cows from both treatment groups (control, zinc) and time points (enrollment, calving) are in green. Calves born to dams enrolled in the feeding trial are yellow. (**D**) Linear mixed effect models were used to compare the top 50 most abundant ARGs in cows and calves using log_2_ transformed RPKM values. Multiple tests were adjusted for using Benjamini-Hochberg method. Yellow dots (negative values) indicate genes that were significantly enriched (*p* < 0.05) in calves relative to cows while green dots (positive values) are ARGs that were significantly enriched in cows. Gray dots represent RPKM values not significantly different between groups. (**E**) Heatmap depicting Pearson’s product minute correlation coefficient between the relative abundance of bacterial taxa and ARG abundance (RPKM). Residuals from linear model regressions of taxa and ARG abundance were used for correlation analysis. Taxa with > 1% mean relative abundance and ARGs that were found in 99% of cow and calf samples were included. Diamonds indicate ARGs that were significantly enriched in calves relative to cows (yellow), enriched in cows relative to calves (green), or were found in similar levels between populations (gray) as depicted in (**D**). Boxes with asterisk indicate significant correlations after false discovery rate correction (*p* < 0.05)

Metagenomic analysis of the calf resistome found 192 ARGs present in at least 90% of neonatal calf samples. Despite the cow and calf microbiomes having significantly different compositions and overall bacterial abundances (Figs. 4), 115 (60%) of 192 ARGs present in calves were also found in cows. As with the cows, the calf ARGs mostly conferred resistance to multiple classes of antimicrobials, with the most abundant being tetracyclines, glycopeptides, and streptogramins. Sixteen of 17 *tet*-family genes and 26 of 29 glycopeptide resistance genes were shared between offspring and dam. There was no significant difference in ARG abundances between calf treatment groups. Thirty-two of the 50 most abundant ARGs found in both calves and cows were found at significantly higher abundances in calves than in cows (Fig. 4D).

Correlation analysis of bacterial taxa and ARG abundances was performed on the most abundant ARGs that were shared between cow and calf samples (Fig. 4E, Supplemental Fig. 3). Many of the ARGs that were significantly enriched in calf samples (yellow diamonds) relative to cows correlated strongly with *E. coli* and the Enterobacteriaceae family, taxa that dominated the early calf microbiome. ARGs that were enriched in cow samples (green diamonds) correlated with a different, diverse set of bacteria taxa including *Bifidobacterium pseudolongum*, *Methanobrevibacter* sp., *Ruminococcus* sp., and the Clostridiales order (Fig. 4E, Supplemental Fig. 3). Correlation coefficients in cow samples were less robust than in calves due to the increased complexity of the microbiome. However, these findings support early gut colonizers contribute to the diverse and abundant calf resistome.

## Discussion

In this first study to explore the effect of high levels of dietary zinc in gestating cows on the cow and calf microbiome and resistome, we found no significant differences in the gut microbiota of cows or their calves fed normal or high levels of dietary zinc, and minimal differences in the distribution of ARGs. In contrast, substantial changes in the diversity and composition of the microbiota and of ARGs were observed between enrollment (~ 6 weeks prior to calving) and calving, or throughout the dry-off period. Our findings are consistent with what has been seen in other studies [24–26], where marked changes in the composition of the rumen microbiome were observed during the transition period, including increases in the relative abundance of Proteobacteria and decreases in levels of Firmicutes. The transition period is characterized by substantial physiological and metabolic changes, including inflammation and increases in gut permeability [27, 28], and intake usually drops precipitously a day or two before calving. In this cohort of cows, the average consumption decreased from 30 lbs dry matter in the far-off period (i.e., at enrollment) to 23 lbs in the few days prior to calving. These changes are reflected in the altered microbiota observed in this study, including changes in levels of ARGs. While it is impossible to directly link ARG with bacterial origins without long-read metagenomic sequencing, many studies have shown that changes in the composition of the microbiota, driven partially by diet, drive changes in the resistome [29–31]. Many of these same studies also found that on-farm antimicrobial use altered the resistome. Multiparous cows at our facility were all treated with intramammary dry cow therapy (penicillin and dihydrostreptomycin) during the dry period. Interestingly though, we saw increases in only two ARGs in both treatment and control groups at calving that conferred resistance to streptogramin antibiotics (*vgaC* [32] and *vgaE* [33]), and none that conferred resistance to penicillins. The effects of dry cow therapy on the milk microbiome and resistome have been shown to vary. Some investigators found no effect on ARG abundance in milk [34] or feces [35], while others saw delayed effects in the milk 2–6 months after calving [36].

Higher levels of dietary zinc impacted only a small number of taxa and ARGs in the gestating cows, including *Faecalibacterium*, *Bacteroides*, *Turicibacter, Bifidobacterium pseudolongum*, and the ARGs *arlS, cdeA, cfrE, dfrA3, lsaC, lsaE, macB, msrA, msrC, pmrE, tet(37), tet(A)* and *vanRF*. In calves, *Faecalibacterium* is an important genus that contributes to the development and health of the gastrointestinal tract by metabolizing acetate to butyrate, a volatile fatty acid that fuels and stimulates the growth and differentiation of colonocytes [37, 38]. Higher relative abundances of *Faecalibacterium* are associated with increased weight gain and a reduced likelihood of diarrhea in calves [39]. The role of *Faecalibacterium* in the adult cow GI tract is not well understood. One study found that ruminal *Faecalibacterium* levels in transition cows were negatively associated with milk production [40], while another study found that cows administered probiotics had higher levels of *Faecalibacterium* [41]. *Bacteroides* are rumen fermentative bacteria that tend to be one of the dominant genera in the bovine intestine [42]. The role of *Bacteroides* in gut health of adult dairy cows is conflicting, with one study finding increased levels of *Bacteroides* in cows fed probiotics [41] and another finding increased levels in cows treated with cephalosporins [43].

The antimicrobial resistance genes that were increased in the treatment cows at calving conferred resistance to multiple classes of antimicrobials (*cdeA*) as well as to specific classes such as tetracyclines (*tet(37)*, *tet(A*)), lincosamides (*lsaC, lsaE*), trimethoprim (*dfrA3*), macrolides (*macB*), polymyxins (*pmrE*) and chloramphenicol (*cfrE*). The selection of some of these ARGs by dietary zinc, especially those involved in efflux mechanisms (e.g., *cdeA, macB, msrA, msrC, pmrE*), can partially be explained by the fact that they also confer cross-resistance to metals, as both antimicrobials and heavy metals are excreted by the same efflux pump systems [44]. Other studies in swine and beef and dairy cattle have also found that dietary zinc selects for resistance to resistance to trimethoprim, macrolides, and lincosamides [8, 9, 45–47].

The lack of an overall strong effect of dietary zinc on the microbiota and resistome of dairy cows is consistent with findings from other studies. Lactating dairy cattle supplemented with zinc experienced no change in richness, diversity, or composition of the gut microbiota [16, 48]. In growing beef cattle supplemented with dietary zinc, a similar stability in the overall composition of the microbiota was seen, with some shifts in relative abundance of certain phyla (i.e., increases in *Firmicutes, Tenericutes* and *Actinobacteria*) [49]. Similarly, only a small number of ARGs were found to be increased in studies with high levels of dietary zinc in pigs [45] and beef cattle [50, 51]. It is unclear why such minimal effects were observed in our dairy cows. Homeostatic mechanisms in cows ensure that excess dietary zinc is rapidly excreted [52, 53]; and while some uptake by ruminal microbes may occur [54], most of the free zinc transits through the large intestine where it is expected to be accessible to and act upon the colonic microbiota. Marked changes in the gut microbiota have been reported in other species fed high levels of dietary zinc [11], including mice [55], pigs [56–59], poultry [60] and horses [61]. Differences in the structure of these species’ gastrointestinal tracts (e.g., monogastric vs. ruminant) may partly explain the differences in observed impact on the microbiota. Differences in the amount, type, and route of exposure of the dietary zinc may also explain the degree of observed changes. Indeed, one study found that long-term exposure to high levels of zinc in contaminated drinking water resulted in significant and sustained changes in the resistome of dairy cattle [9]. Our results suggest that dietary zinc at the levels included in our study and fed for a short duration (6 weeks or less) have a relatively negligible effect on the gut microbiota and resistome. Indeed, the modest increase in levels of potentially beneficial commensals (i.e., *Bifidobacteria, Turicibacter, Faecalibacteria* and *Bacteroides*) and the observed trend of lower levels of shared antimicrobial resistance genes in treatment cows compared to control cows, even if not statistically significant, suggests that dietary zinc is unlikely to be detrimental to the gut microbiota and may even be favorable in certain circumstances.

The lack of any observed effect of the dam’s treatment on the neonatal calf microbiota and resistome may represent a similar effect as that seen for the cow microbiota. However, it may also reflect an overall immaturity of the gut microbiota. Neonatal calves have a simple, less diverse microbiota that increases in complexity and diversity with growth [20]; at less than 3 days of age, the influences of external factors such as the dam’s treatment on the neonatal microbiota may not be strong enough to detect. Additionally, because calves at our barn rarely receive their own dam’s colostrum but more often receive pooled stored colostrum, the effect of the dam is even less marked. In another study, dietary zinc had minimal impact on the diversity or richness of the gut microbiota in neonatal calves, though the balance of certain genera shifted (i.e., increase in *Bacteroidetes, Lactobacillus* and *Faecalibacterium*) [16], which is consistent with what we observed.

We did find that calves had higher levels of certain ARGs than cows, most of which confer resistance to multiple classes of antimicrobials, are related to drug efflux pumps [62, 63], and are often found in *E. coli* or *Enterococcus*, which are 2 taxa encountered at high levels in calves. Dairy calves in general have a more rich and diverse resistome than cows [64], and the resistance determinants we detected are often found in dairy calves, especially in the youngest calves and those fed waste milk containing antimicrobial residues [65–68]. Calves in this study were all neonates (< 3 days of age) that received pooled colostrum and waste milk, including from cows treated with antimicrobials. A previous study found that 90% of ARGs found in calves at 2 days of age were also present in colostrum, suggesting colostrum is a large driver of early resistome establishment [69]. The lack of effect of the dam’s zinc regimen on distribution of ARGs in the calves is therefore understandable, as other factors are more important determinants. However, the fact that 60% of ARGs found in calves were also found in cows does suggest some maternal contribution to the pool of ARGs in calves.

## Conclusion

Marked changes in the microbiota of gestating cows were seen during the drying off period, with decreased alpha diversity and shifts in the composition of the microbiota at calving relative to drying off. However, the impact of high levels of dietary zinc was minimal, with no observed changes in alpha or beta diversity, and changes in the relative abundance of only a small number of taxa and ARGs. Maternal dietary zinc had no significant effects on the microbiota or resistome of neonatal calves. As noted previously [18], there appears to be a wide margin of safety in zinc levels both on the cow and its gut microbiota.

### Electronic supplementary material

Below is the link to the electronic supplementary material.

Supplementary Material 1

Supplementary Material 2

Supplementary Material 3

Supplementary Material 4

## Data Availability

The fecal bacterial 16S rRNA sequences supporting the findings of this study are available at the National Center for Biotechnology Information Sequence Read Archive (NCBI SRA) under the accession number PRJNA1066879. Metagenomic sequencing data has been deposited in the same database under Accession Number PRJNA1088536.
