# Supplementary figures and images for: Effects of dietary zinc on the gut microbiome and resistome of the gestating cow and neonatal calf
Source: Anim Microbiome. 2024 Jul 19;6:39. doi: 10.1186/s42523-024-00326-3 (PMC11264502; doi:10.1186/s42523-024-00326-3)

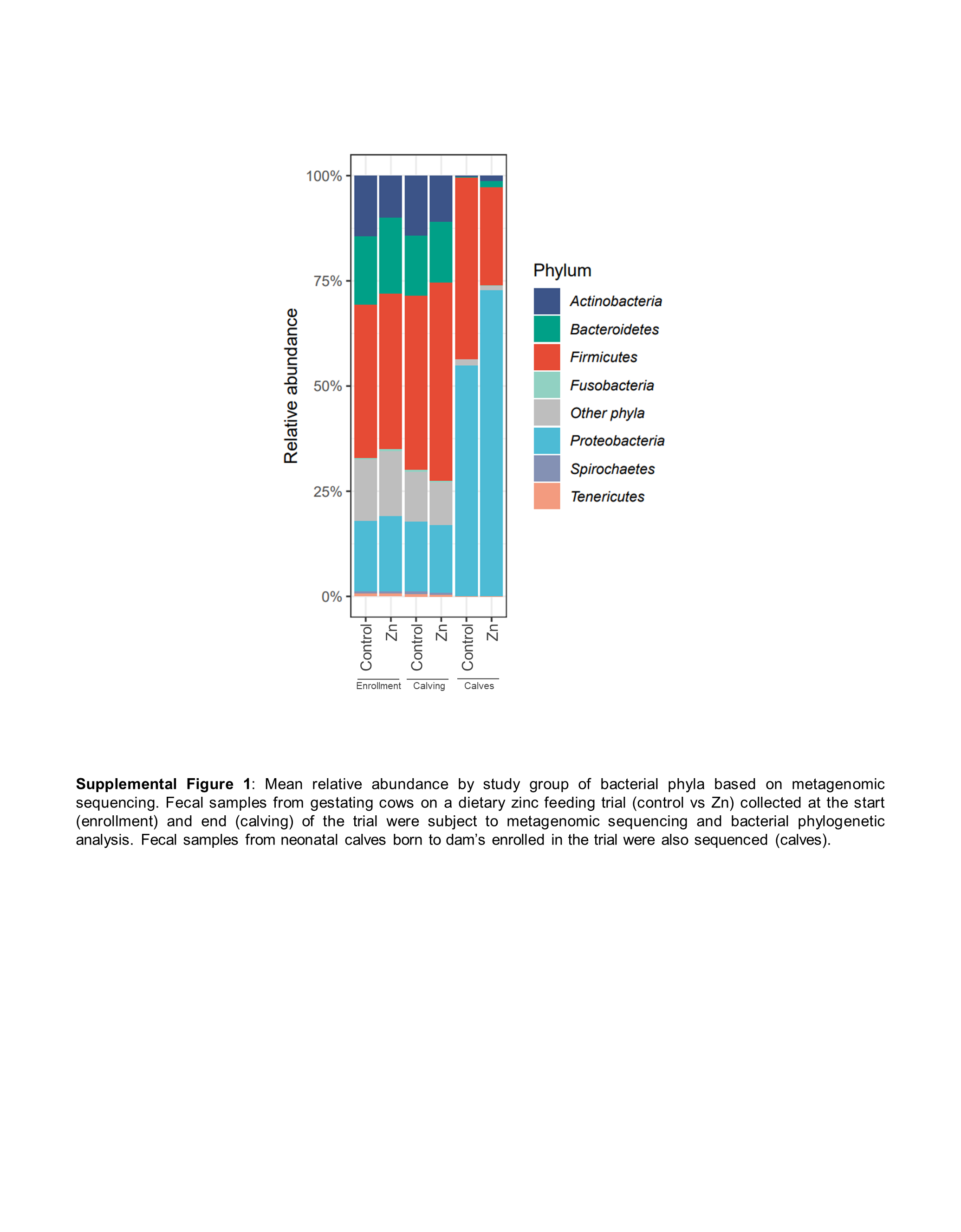

Supplement: Supplementary file 1 — Supplementary Material 1 [file 42523_2024_326_MOESM1_ESM.png]

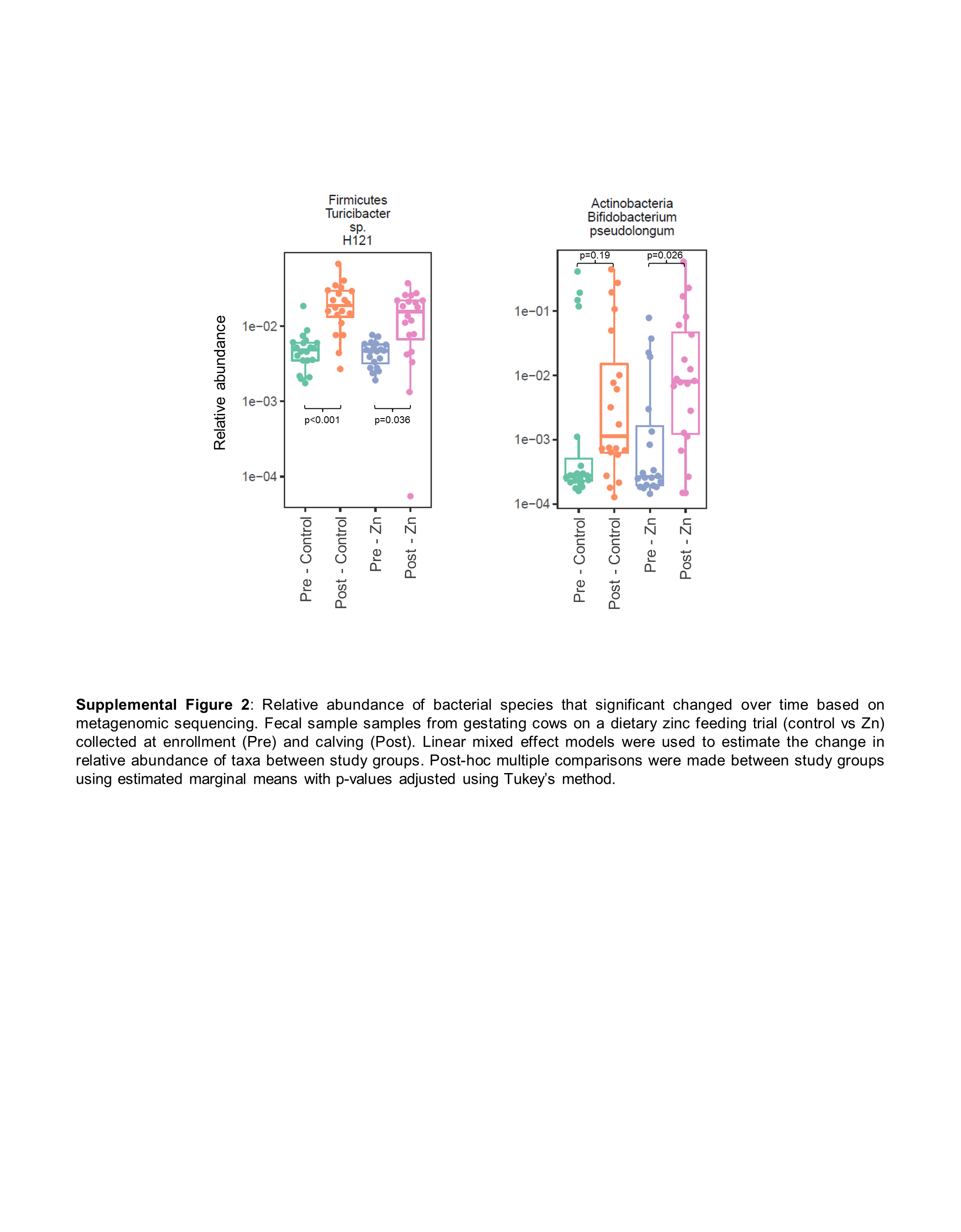

Supplement: Supplementary file 2 — Supplementary Material 2 [file 42523_2024_326_MOESM2_ESM.png]

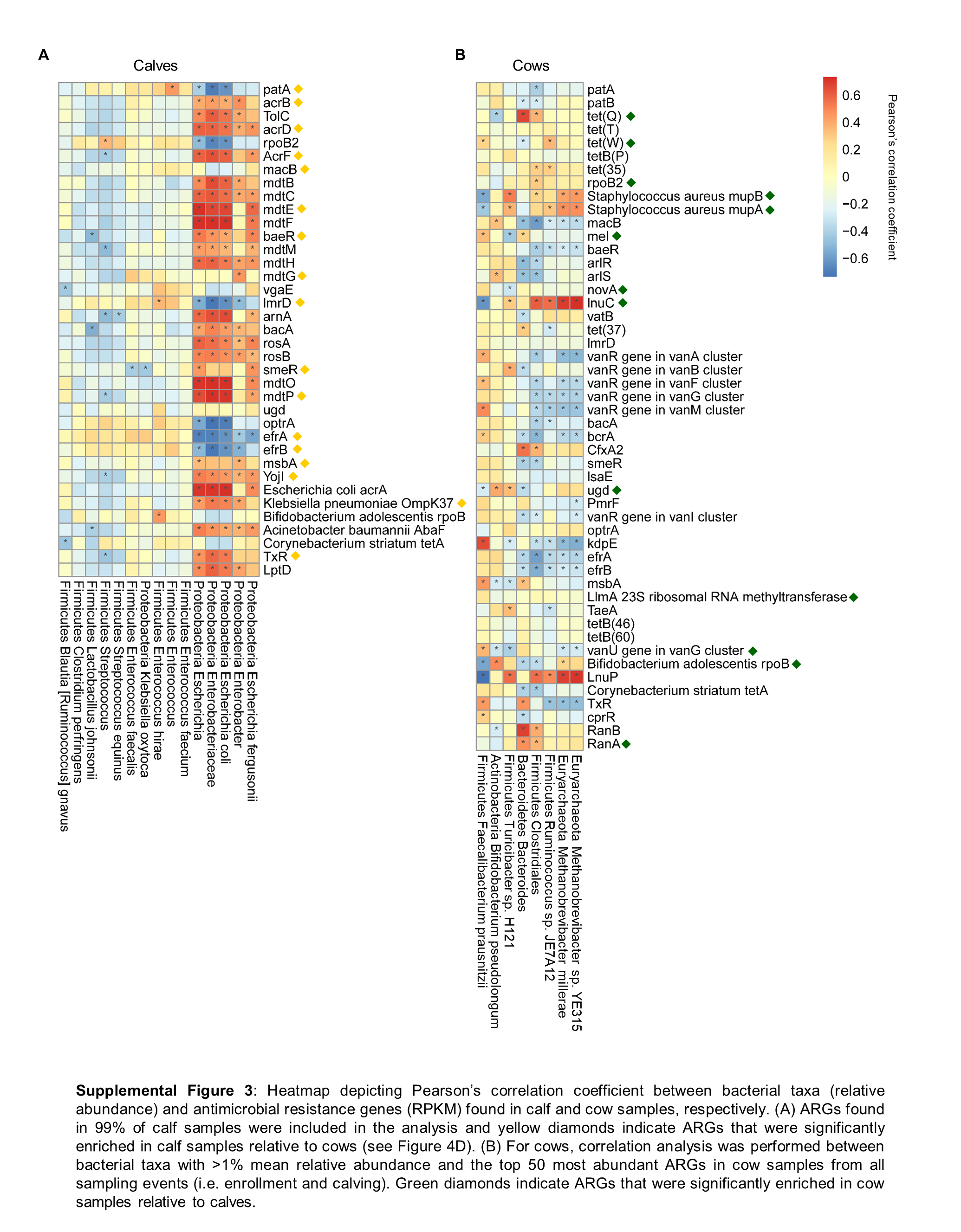

Supplement: Supplementary file 3 — Supplementary Material 3 [file 42523_2024_326_MOESM3_ESM.png]
