# Supplementary material for: Effects of dietary zinc on the gut microbiome and resistome of the gestating cow and neonatal calf
Source: Anim Microbiome. 2024 Jul 19;6:39. doi: 10.1186/s42523-024-00326-3 (PMC11264502; doi:10.1186/s42523-024-00326-3)
Supplement: Supplementary file 4 — Supplementary Material 4 [file 42523_2024_326_MOESM4_ESM.pdf]

Supplemental Table 1: Control and Zinc Treatment Diets Formulated for 11.45 Kg/d

**Diet Formulation (Kg/d)**	**Control**	**Treatment**	
Corn Silage	5.1	5.1	
Grass Hay	3.4	3.4	
Triticale	1.0	1.0	
Concentrate Mix[1]	1.9	1.9	
Corn Distillers Grain	0.113	-----	1
Corn Distillers Grain + ZnCl[2]	-----	.113	

[1] Concentrate mix: CP 53.9%, NDF 11.3%, NFC 18.0%, Fat 1.1%, Ash 15.0%, Ca 0.61%, P 0.53%, Mg 2.2%, K 1.7%, S 1.45%, Na 0.67%, Cl 1.05% DM; Fe 505.0, Cu 24.7, Mn 71.6, Zn 117.9, Se 1.83 PPM. Vitamin A 55.8 KIU/Kg, Vitamin D 14.0 KIU/Kg, Vitamin E 195.0 IU/Kg.

[2] Distillers and ZnCl Treatment: 97.3% Corn Distillers and 2.7% Zinc Hydroxy-chloride.

Supplemental Table 2: Nutrient Content of Control and Zinc Treatment Diets

**Diet Specifications (%DM)**	**Control**	**Treatment**
CP	15.9	15.9
ADF	26.7	26.7
NDF	44.0	43.9
NFC	31.1	31.1
Fat	2.4	2.4
Ash	6.7	6.7
Macro-Minerals		
Ca	0.36	0.36
P	0.26	0.26
Mg	0.51	0.51
K	1.39	1.39
S	0.44	0.44
Na	0.15	0.15
Cl	0.65	0.67
Trace Minerals (PPM)		
Fe	260	260
Mn	54	54
Cu	11	11
Zn	41	205
